# A Guide for Sparse PCA: Model Comparison and Applications

**DOI:** 10.1007/s11336-021-09773-2

**Published:** 2021-06-29

**Authors:** Rosember Guerra-Urzola, Katrijn Van Deun, Juan C. Vera, Klaas Sijtsma

**Affiliations:** 1 Department of Methodology and Statistics, Tilburg University, Prof. Cobbenhagenlaan 225, Simon Building, Room S 820, 5037 DB Tilburg, The Netherlands; 2 Department of Methodology and Statistics, Tilburg University, Tilburg, The Netherlands; 3 Department of Econometrics and OR, Tilburg University, Tilburg, Netherlands; 4 Tilburg University, Tilburg, The Netherlands

**Keywords:** dimension reduction, exploratory data analysis, high dimension-low sample size, regularization, sparse principal components analysis

## Abstract

PCA is a popular tool for exploring and summarizing multivariate data, especially those consisting of many variables. PCA, however, is often not simple to interpret, as the components are a linear combination of the variables. To address this issue, numerous methods have been proposed to sparsify the nonzero coefficients in the components, including rotation-thresholding methods and, more recently, PCA methods subject to sparsity inducing penalties or constraints. Here, we offer guidelines on how to choose among the different sparse PCA methods. Current literature misses clear guidance on the properties and performance of the different sparse PCA methods, often relying on the misconception that the equivalence of the formulations for ordinary PCA also holds for sparse PCA. To guide potential users of sparse PCA methods, we first discuss several popular sparse PCA methods in terms of where the sparseness is imposed on the loadings or on the weights, assumed model, and optimization criterion used to impose sparseness. Second, using an extensive simulation study, we assess each of these methods by means of performance measures such as squared relative error, misidentification rate, and percentage of explained variance for several data generating models and conditions for the population model. Finally, two examples using empirical data are considered.

**Supplementary Information:**

The online version contains supplementary material available at 10.1007/s11336-021-09773-2.

Principal component analysis (PCA) is one of the oldest and most popular multivariate analysis techniques used to summarize a (large) set of variables in low dimension with minimum loss of information (Jolliffe and Cadima 2016; Wold et al. 1987). In particular, PCA is one of the most popular techniques used to analyze (ultra-) high-dimensional data consisting of many more variables than observations, and its use has become more widespread over recent years. PCA is mainly used to summarize the individual variables’ scores by a few derived components based on a linear combination of the individual variables. These new variables are known as component scores and are often used as a data pre-processing step to deal with a large number of variables, e.g., to reduce the number of predictor variables to account for collinearity issues in regression analysis. The coefficients of the linear combination, used to derive the component scores, are known as component weights (Adachi and Trendafilov 2016). Additionally, PCA can give insight into the data structure via the correlation between component scores and variables. These correlations are known as component loadings.

In PCA, there is a long-standing tradition to look for sparse representations where the variables are associated with only one or a few components (Kaiser 1958). The sparse structure facilitates interpretation, and the need for such a representation is especially warranted in the case of an extensive collection of variables. Moreover, sparse representations have been employed not only for interpretational issues but also to deal with the inconsistency of the estimated component loadings/weights in the high-dimensional setting (Johnstone and Lu 2009).

There is a substantial volume of work in sparse PCA based on the different formulations of PCA and using different approaches to achieve sparsity. We categorize sparse PCA methods by their estimation aim: sparse loadings or sparse weights. To obtain sparse loadings, Kaiser (1958), Jolliffe (1995), Cadima and Jolliffe (1995), and Kiers (1994) used a rotation of the PCA solution to obtain a simple structure, and Shen and Huang (2008), and Papailiopoulos et al. (2013) introduced a least-squares low-rank approximation with sparsity inducing penalties such as the lasso (Tibshirani 2011). For sparse weights, Jolliffe et al. (2003) modified the original PCA problem to satisfy the lasso penalty (SCoTLASS), while Zou et al. (2006) used a lasso penalized least-squares approach to obtain sparsity. d’Aspremont et al. (2007b) and d’Aspremont et al. (2007a) established a sparse PCA method subject to a cardinality constraint based on semidefinite programming (SDP), while Journée et al. (2010) and Yuan and Zhang (2013) introduced variations of the well-known power method to achieve sparse PCA solutions using sparsity inducing penalties.

Most of the formulations for sparse PCA are based on different formulation of PCA; thus, the corresponding optimization problems solved are different and—unlike ordinary PCA—do not yield equivalent solutions. Importantly, the different methods result in sparse estimates for different model structures. Hence, the selected method should depend on the objective of the analysis and the assumed model structure for which sparsity is desired. These differences in sparse PCA formulations have remained mostly unnoticed in the literature, which highlights the need for a thorough comparison of the methods under different data generating models—imposing sparsity on different model structures—and concerning different performance measures. The objective of our research is to provide a guide for using sparse PCA, emphasizing the differences in purposes, objectives, and performance among several sparse PCA approaches. We present a review of the most relevant sparse PCA methods used for sparse loadings and sparse weights estimation. We assess these methods by conducting an extensive simulation study using three types of sparse data structures and performance measures such as squared relative error, misidentification rate, and percentage of explained variance. Finally, we use two empirical data sets to illustrate how to use these methods in practice. The data sets consist of item scores on a questionnaire measuring the Big Five personality (Dolan et al. 2009) and gene expression profiles of lymphoblastoid cells used to distinguish different forms of autism ( Nishimura et al. 2007). The former example relies on questionnaire data for which researchers wish to understand the correlation patterns in the data (e.g., knowing which items are highly correlating and hinting at an underlying component or construct). In contrast, the latter example relies on high-dimensional data collected in a classification setting where a reduction of the large set of variables is performed as a pre-processing step.^1^ Results from the simulation study and empirical applications suggest that sparse loadings methods are more suitable for exploratory data analysis, while sparse weights methods are more suitable for summarization.

The paper is organized as follows. Section 1 describes different approaches and drawbacks of PCA. In Sect. 2, the leading methods for sparse PCA are briefly discussed. Simulation studies are presented in Sect. 3, and two examples using empirical data sets are presented in Sect. 4. Concluding remarks are made in Sect. 5. Next, we collect our notation for our readers’ convenience.

*Notation* Matrices are denoted by bold uppercase, the transpose of a matrix by the superscript $^\top$ (e.g., ${\mathbf{A}}^{\top }$), vectors by bold lowercase, and scalars by lowercase italics, and we will use capital letters (of the letter used to run an index) to denote cardinality (e.g., *j* running from 1 to *J*). Given a vector $\mathbf{x}\in {\mathbb {R}}^{J}$, its *j*-th entry is denoted by $x_j$. The $l_0$-norm $\left\| \mathbf{x}\right\| _{0}$ is the number of nonzero elements of $\mathbf{x}$, the $l_1$-norm is defined by $\left\| \mathbf{x}\right\| _{1} = \sum _{j=1}^{J} \left| x_j \right|$, and the Euclidean distance by $\left\| \mathbf{x}\right\| = (\sum _{j=1}^{J} x_{j}^{2})^{1/2}$. Given a matrix $\mathbf{X}\in {\mathbb {R}}^{I\times J}$, its *i*-th row and *j*-th column entry is denoted by $x_{i,j}$, $\left\| \mathbf{X}\right\| _{F}^{2}=\sum _{i=1}^{I}\sum _{j=1}^{J}\left| x_{i,j} \right| ^{2}$ denotes the squared Frobenius norm, and $Tr(\mathbf{X})=\sum _{i=1}^{I} x_{i,i}$ denotes the trace operator when $\mathbf{X}$ is square matrix ($I=J$). We use the notation $\mathbf{X}_K \in {\mathbb {R}}^{I\times K}$, with $K<J$, for the matrix whose columns are the first *K* columns of $\mathbf{X}$. Given a scalar $\delta \in {\mathbb {R}}$, $\left[ \delta \right] _{+}=\max (0,\delta )$. The soft-thresholding operator is defined as $(S(x,\lambda )=\text {sign}(x)[ |x|-\lambda ]_{+})$, where sign denotes the sign of *x*. Finally, when formulating an optimization problem, s.t means “subject to”.

## Principal Component Analysis Overview

This section aims to review different formulations for PCA and their relation to the singular value decomposition (SVD) and the eigenvalue decomposition (EVD). PCA formulations are presented in Sect. 1.1. Section 1.2 discusses the lack of consistency in the estimation of the component loadings/weights and the difficulties to interpret the component scores—the main drawbacks of PCA found in the literature. Let us define $\mathbf{X}\in {\mathbb {R}}^{I\times J}$ as the data matrix (i.e., *I* observations and *J* variables) and $K<J$ the number of desired components. Without loss of generality, we follow the common practice of assuming that all the data are centered and scaled to unit variance, that is $\mathbf{X}^{\top }{} \mathbf{1}_{I}= \mathbf{0}_{J}$ and  denotes the sample correlation matrix (Jolliffe and Cadima 2016).

### PCA Formulations

Several disciplines rely on the following structure for the data set (Whittle 1952),

1 $$\begin{aligned} \mathbf{X = TP}^{\top }+\mathbf{E }, \end{aligned}$$

where $\mathbf{T}\in {\mathbb {R}}^{I\times K}$, $\mathbf{P}\in {\mathbb {R}}^{J\times K}$, $\mathbf{P}^{\top }{} \mathbf{P}= \mathbf{I}\in {\mathbb {R}}^{K\times K}$, $\mathbf{I}$ denotes de identity matrix, and $\mathbf{E}\in {\mathbb {R}}^{I\times J}$ is the error matrix uncorrelated to $\mathbf{TP^{\top }}$. $\mathbf{P}$ is called the component loadings matrix and $p_{j,k}$ are the component loadings, which express the strength of the connection between the variables and the component scores $\mathbf{T}$. In this model, the component scores are linear combinations of the original variables; therefore, they can be expressed as $\mathbf{T} = XW$, where the elements $w_{j,k}$ express the weights used in this combination. The elements of the matrix $\mathbf{W}\in {\mathbb {R}}^{J\times K}$ are named component weights. For this approach, the goal of PCA is to minimize the squared Frobenius norm of the error matrix $\mathbf{E}$ (also known as the least-squares approach). The problem is formulated as:

2 $$\begin{aligned} ({\widehat{\mathbf{T}},\widehat{\mathbf {P}}})=\mathop {{\mathrm{argmin}}}\limits _{\mathbf{T,P}}&\left\| \mathbf{X-TP}^{\top }\right\| _{F}^{2} \nonumber \\ \text {s.t.}&\mathbf{P}^{\top }{} \mathbf{P= I}. \end{aligned}$$

A solution of problem (2) can be obtained from the truncated SVD of $\mathbf{X=UDV}^{\top }$, with $\mathbf{U}\in {\mathbb {R}}^{I\times K}$ and $\mathbf{V}\in {\mathbb {R}}^{J\times K}$ semi-orthogonal matrices such that $\mathbf{U^\top U=V^\top V=I}\in {\mathbb {R}}^{K\times K}$ and $\mathbf{D}\in {\mathbb {R}}^{K\times K}$ a diagonal matrix (Eckart and Young 1936). Thus, ${\widehat{\mathbf{T}} = \mathbf {U}}{} \mathbf{D}$ and ${\widehat{\mathbf{P}} = \mathbf {V}}$ provide the solution of problem (2).

In psychometrics, it is common to find PCA formulations, where problem (2) is modified as follows (ten Berge 1986),

3 $$\begin{aligned} ({\widehat{\mathbf{T}},\widehat{\mathbf {P}}})=\mathop {{\mathrm{argmin}}}\limits _{\mathbf{T,P}}&\left\| \mathbf{X-TP}^{\top }\right\| _{F}^{2} \nonumber \\ \text {s.t.}&\mathbf{T}^{\top }{} \mathbf{T}= (I-1){\mathbf{I}}. \end{aligned}$$

The solution of problem (3) can be obtained using the SVD of $\mathbf{X}$ by taking ${\widehat{\mathbf{T}}} = (I-1)^{1/2}{} \mathbf{U}$ and ${\widehat{\mathbf{P}}} = (I-1)^{-1/2}\mathbf{V}{} \mathbf{D}$.^2^ Hence,

$$\begin{aligned} {\widehat{\mathbf {T}}}&= \mathbf {(X-E)}\mathbf {P} (\mathbf {P}^{\top }\mathbf {P})^{-1}\\ {}&= (I-1)^{1/2}{} \mathbf {X}{} \mathbf {V} {} \mathbf {D}^{-1}. \end{aligned}$$

Therefore, the component weights matrix for problem (3) is ${\widehat{\mathbf {W}}} = (I-1)^{1/2} {} \mathbf {V}{} \mathbf {D}^{-1}$. Additionally, problem (3) is commonly formulated as an explicit combination of the original variables (ten Berge 1993), considering $\mathbf{T} = XW$ that is

$$\begin{aligned} ({\widehat{\mathbf{W}},\widehat{\mathbf {P}}})=\mathop {{\mathrm{argmin}}}\limits _{\mathbf{W,P}}&\left\| \mathbf{X-XWP}^{\top }\right\| _{F}^{2} \nonumber \\ \text {s.t.}&\mathbf{T}^{\top }{} \mathbf{T}= (I-1)\mathbf{I}. \end{aligned}$$

The classical way to define PCA is to find the component weight matrix $\mathbf{W}\in {\mathbb {R}}^{ J\times K}$, having orthogonal vectors that maximize the variance of the components. Formally, consider the following formulation:4A solution for problem (4) can be obtained from the EVD (Hotelling 1933) of the covariance matrix , taking $\widehat{\mathbf{W}} = \mathbf {V}$ as the matrix formed by eigenvectors corresponding the *K* largest eigenvalues.

The orthogonality constraints in PCA formulations (2) and (4) and principal axes orientation imply their equivalence. More precisely, component loadings and component weights are both equal to $\mathbf{V}$. To see this, notice that using the SVD of $\mathbf{X} = \mathbf{UDV}^T$, the EVD for is obtained (Jolliffe and Cadima 2016). Thus, $\mathbf{D}^{2}$ is the diagonal matrix containing the eigenvalues of  (the square of the singular values of $\mathbf{X}$) in decreasing order: $d^{2}_{11}\ge d^{2}_{22} \ge \ldots \ge d^{2}_{JJ}$. Then, the matrix of component weights ${\widehat{\mathbf{W}} = \mathbf {V} }$ coincides with the matrix ${\widehat{\mathbf{P}}}$ of component loadings defined by PCA formulation (2). However, this equivalence does not hold exactly for PCA formulation (3) because the orthogonality constraint is imposed on the component scores. Instead, under formulation (3), $\widehat{\mathbf{W}}$ and ${\widehat{\mathbf{P}}}$ are proportional to $\mathbf{V}$.

### PCA Drawbacks

#### Interpretation and Non-uniqueness

Principal component scores are a linear combination of the original variables. That makes them difficult to interpret. For instance, when using data containing measures with different units, the linear combination does not have a definite meaning. A common practice to tackle this problem is to use the correlation matrix instead of the covariance matrix (Jolliffe and Cadima 2016). That is to standardize the variables, so all of them are on the same scale.

Rotation techniques are commonly used to help practitioners interpret the component loadings. The rotation is done to obtain component loadings values close to either 0 or 1, such that only the most relevant variables are considered for interpretation purposes (see Sect. 2.1.1 for further discussion). The rotation can be implemented using an orthogonal rotation matrix $\mathbf{Q}$ which does not modify the amount of variance accounted for by all components together but rather redistributes the variance across the variables by choosing a different system of orthogonal axes. However, because of the several possible choices for the rotation matrix $\mathbf{Q}$, non-unique solutions in problems (2) and (4) are achieved (Hastie et al. 2000).

#### Inconsistency in the High-Dimensional Setting

As mentioned above, the solution of the model-free PCA formulation (4) is the leading eigenvector of the covariance matrix. Inconsistency of this leading eigenvector has been studied analyzing the angle between its population and estimated value, under different asymptotical conditions for the dimensionality of the data set. For instance, Johnstone and Lu (2009) show that

$$\begin{aligned} P\left( \lim \limits _{{I\rightarrow \infty }}R^2 (\hat{\mathbf{v}}_1,\mathbf{v}_1) = R_{\infty }^{2}(\omega ,c) \right) = 1, \end{aligned}$$

where $\mathbf{v}_1$ is the leading population eigenvector, $\hat{\mathbf{v}}_1$ its estimate, and $R^2 ({\hat{v}}_1,v_1)$ the cosine of the angle between $\hat{\mathbf{v}}_1$ and $\mathbf{v}_1$. $\omega >0$ stands for the limiting signal-to-noise ratio, $c =\lim \limits _{{I\rightarrow \infty }} J/I$, and $R_{\infty }^{2} = (\omega ^{2}-c )_{+}/(\omega ^{2}+c\omega )$. This result implies that $\hat{\mathbf{v}}_1$ is a consistent estimate of $\mathbf{v}_1$ if and only if $c=0$. Therefore, in the high-dimensional setting ($J\gg I$), the estimator of the component weights in the PCA formulation (4) is inconsistent. Similarly, the estimation of the leading eigenvalue is shown to be inconsistent under random matrix theory (e.g., when *I* and *J* tend to infinity and the ratio *I*/*J* converges to a constant) (Baik and Silverstein 2006; Paul 2007; Nadler 2008; Johnstone and Lu 2009) and in the high-dimensional low sample size (HDLSS) (e.g., *J* tends to infinity, and *I* is fixed) (Jung and Marron 2009; Shen et al. 2016a). On the other hand, Jung and Marron (2009) show that, when *I* is fixed, the angle between $\hat{\mathbf{v}}_1$ and $\mathbf{v}_1$ goes to 0 with probability 1 if the leading eigenvalues are extremely large in comparison with the number of variables *J*, yet the components scores are shown to be inconsistent (Shen et al. 2016b).

## Sparse Principal Component Analysis Overview

Sparse PCA has been proposed as a solution to the difficulties encountered in interpreting the component scores of ordinary PCA, non-uniqueness, and the inconsistency of the component loadings/weights (c.f. Sect. 1.2). Research efforts have focused on reformulations for PCA, where component loadings or component weights have as many zero elements as possible. In this section, we present six sparse PCA methods that are well established in the literature and for which implementations are available. Our selection of methods was also chosen to reflect the different PCA formulations (2), (3), and (4). This section aims to show the differences in the purposes and objectives of sparse PCA methods. The emphasis is on the fact that while the ordinary PCA formulations (2) and (4) are equivalent (see Sect. 1.1), for sparse PCA the corresponding formulations are not equivalent, so that the obtained results heavily depend on the chosen methodology. Sparse PCA methods for estimating the loadings are presented in Sect. 2.1, while sparse PCA methods for estimating the weights are presented in 2.2.^3^

### Sparse Loadings

Principal component analysis, when used to explore structure and patterns in data, relies on the model structure presented in Eq. (1). Interpreting the components is based on inspecting the loadings because these reveal how strongly the variables contribute to the components. More precisely, in problem (2), the component loadings $\mathbf{P}$ represent the regression coefficients in the multiple regression of $\mathbf{x}_j$ on the *k* component scores $\mathbf{t}_k$.^4^ Note that with orthogonal component scores this is a regression problem with independent predictors and with proper normalization constraints the loading is equal to the correlation. Then, having sparse component loadings gives a clearer interpretation in the sense that variables are explained only by one or a few components. In this section, we present two frequently used methodologies for this purpose.

#### Sparse PCA Via Rotation and Thresholding: Varimax and Simplimax

The first attempts to achieve a component structure with variables being explained by one component only while having zero loadings for the other components are simple structure rotations followed by thresholding. Simple structure rotation, which was adopted from factor analysis, (Jolliffe 2002, 1995, 1989, Chap. 11), relies on the rotational freedom of Eq. (1):

5 $$\begin{aligned}&\mathbf {X = TP^{\top } + E = T(Q^{-1})^{\top }}{} \mathbf {(PQ)^{\top }}+ \mathbf {E}\nonumber \\&\qquad \mathbf {X = T}_{rotated}\mathbf {P}^{\top }_{rotated} + \mathbf {E} \end{aligned}$$

with $\mathbf{Q}$ a non-singular transformation matrix usually orthogonal (hence $\mathbf{Q}$ is a rotation matrix) or oblique^5^ (Jennrich 2004, 2006).

This approach is applied in two steps. First, the component scores and component loadings are obtained from solving problem (2). Second, a rotation matrix $\mathbf{Q}$ is found by optimizing a criterion that leads to a simple structure of $\mathbf{PQ}$. In this study, we consider two well-known methods: Varimax (Kaiser 1958) that maximizes the variance of the squared component loadings hence encouraging loadings to be as close to either 0 or 1 as possible, and Simplimax (Kiers 1994) that finds an oblique matrix such that the rotated loading matrix comes closest (in the least square sense) to a matrix with (at least) a given number of zero values. Oblique rotation matrices are often used when the component scores are expected to be correlated. The rotated loadings will—in general—not be precisely zero, but in practice, small loadings are neglected (including not printing the value of small loadings in leading software packages such as SPSS), which boils down to treating them as having a zero value (Jolliffe 2002, p.269). This practice is called thresholding and is considered *ad hoc*. Importantly, as discussed by Cadima and Jolliffe (1995), the thresholding approach is misleading in the sense that another subset of variables may better approximate the data as in Eq. (5).

#### Sparse PCA via Regularized SVD: sPCA-rSVD

Taking the close connection between the SVD and PCA as a point of departure, Shen and Huang (2008) proposed a sparse PCA method based on adding a regularization penalty to the least-squares PCA criterion in problem (3). Their so-called sparse PCA via regularized SVD (sPCA-rSVD) method solves the following problem:

6 $$\begin{aligned} ({\hat{\mathbf{t}},\hat{\mathbf {p}}})=\mathop {{\mathrm{argmin}}}\limits _{\mathbf{t,p}}&\left\| \mathbf{X- tp}^{\top }\right\| _{F}^{2}+ {\mathcal {P}}_{\lambda }(\mathbf{p}) \nonumber \\ \text {s.t. }&\left\| \mathbf{t} \right\| _{2}^{2} = 1, \end{aligned}$$

where ${\hat{\mathbf{t}} \hat{\mathbf {p}}}^{\top }$ is the best rank-one approximation of the data matrix $\mathbf{X}$ (Eckart and Young 1936), $\mathbf{t}$ is the first component score vector and $\mathbf{p}$ the corresponding loading vector, and ${\mathcal {P}}_{\lambda }$ is a particular penalty term that imposes sparsity on the component loadings. Three different sparsity inducing penalties are considered in Shen and Huang (2008), including the $l_1$-norm of the loadings also known as the lasso. Problem (6) is used to find the first component score and component loading vectors, the subsequent pairs $({\hat{\mathbf{t}}_{k},\hat{\mathbf {p}}_{k}})$ with $k > 1$ are obtained by solving problem (6) for the residual matrix (i.e., $\mathbf{X}-{\hat{\mathbf{t}}\hat{\mathbf {p}}}^{\top }$). Shen and Huang (2008) solved the problem by alternating between the optimization of $\mathbf{t}$ given $\hat{\mathbf{p}}$ and $\mathbf{p}$ given $\hat{\mathbf{t}}$; they also discuss that the conditional optimization problem of the loadings is separable in the variables. Such separability has two major advantages. First, all loadings can be optimized simultaneously using simple expressions (e.g., soft-thresholding of the inner product of the observed variable and component scores) which implies very efficient computation even in the high-dimensional setting; second, it means that the problem can be solved for a fixed number of zero coefficients. Trendafilov and Adachi (2015) used this advantages to solve the least-squares PCA problem (3) with orthogonal $\mathbf{T}$ for $k > 1$ subject to a cardinality constraint.

### Sparse Weights

In this section, we present different methodologies to estimate the sparse component weights matrix $\mathbf{W}$. Given that the role of $\mathbf{W}$ is to weight the original variables to form $\mathbf{T= XW }$, sparsity is desired on $\mathbf{W}$. In this way, the component scores $\mathbf{T}$ would be summarized by a weighted linear combination of those variables in $\mathbf{X}$ with nonzero elements in $\mathbf{W}$.

#### Sparse PCA Via Elastic Net Regularization: SPCA

One of the most popular methods for PCA with sparse component weights was proposed by Zou et al. (2006). They showed that the component weights^6^ are proportional to the solution of a ridge regression, and sparsity can be attained by adding a lasso penalty. Zou et al. (2006) proposed to solve the following problem

7 $$\begin{aligned} ({\widehat{\mathbf{W}},\widehat{\mathbf {P}}})=\mathop {{\mathrm{argmin}}}\limits _{\mathbf{W,P}}&\left\| \mathbf{X- XWP}^{\top }\right\| _{F}^{2}+ \sum _{k=1}^{K} \lambda \left\| \mathbf{w}_{k} \right\| ^{2}+ \sum _{k=1}^{K}\lambda _{1,k} \left\| \mathbf{w}_k \right\| _{1} \nonumber \\ \text {s.t. }&\mathbf{P}^{\top }{} \mathbf{P = I}. \end{aligned}$$

The terms $\sum _{k=1}^{K} \lambda \left\| \mathbf{w}_{k} \right\| ^{2}$ and $\sum _{k=1}^{K}\lambda _{1,k} \left\| \mathbf{w}_k \right\| _{1}$ are the ridge and lasso penalties, respectively. To solve the problem (7) for given values of $\lambda$ and $\lambda _{1,k}$, Zou et al. (2006) proposed an alternating minimization algorithm, that updates $\mathbf{W}$ and $\mathbf{P}$ alternately with the other variable is fixed to its current estimate until some stopping criterion is reached. The update of $\mathbf{P}$ conditional upon fixed $\mathbf{W}$ is the orthogonal Procrustes rotation problem with known optimal solution (Golub and Van Loan 2013). The conditional update of the weights $\mathbf{W}$ can be written as an elastic net regression problem that regresses the component scores ${\mathbf{t}}_k$ on the *J* variables ${\mathbf{x}}_j$ (Zou and Hastie 2005). Note that in the high-dimensional setting, this becomes a high-dimensional regression problem with known numerical issues (Hastie et al. 2001). Then, as the lasso penalty yields at most *I* nonzero coefficients, in the high-dimensional setting the ridge penalty is included. Efficient procedures have been proposed for the elastic net regression problem such as the LARS-EN (Tibshirani et al. 2004), cyclic coordinate descent (Friedman et al. 2007), and proximal gradient techniques (Beck and Teboulle 2009). However, these algorithms remain subject to computational issues in the high-dimensional setting (Yuan et al. 2011). Furthermore, a major challenge when using the elastic net method is a proper tuning of the penalties. In this respect, the LARS-EN algorithm has the benefit that it allows defining the number of nonzero values a priori.

#### Sparse PCA Via Cardinality Penalty: pathSPCA

d’Aspremont et al. (2007a) focused on the problem of maximizing the variance of the components with a cardinality penalty,

8 $$\begin{aligned} \hat{\mathbf{w}}= \mathop {{\mathrm{argmax}}}\limits _{{\left\| \mathbf{w} \right\| \le 1}} \left\| \mathbf{Xw} \right\| ^2-\rho \left\| \mathbf{w}\right\| _{0}, \end{aligned}$$

with $$\rho $$ a parameter controlling the sparsity. d’Aspremont et al. (2007a) proposed a greedy algorithm that provides candidate indexes $$ I_r$$ for *r* nonzero elements. Then the sparse component weights vector is the solution of the problem (8) given $$ I_r$$, which is:

$$\begin{aligned} \hat{\mathbf{w}}= \mathop {{\mathrm{argmax}}}\limits _{{\left\{ \mathbf{w}_{I_{r}^{c}}=0,\, \left\| \mathbf{w} \right\| = 1\right\} }} \left\| \mathbf{Xw} \right\| ^2-\rho r, \end{aligned}$$

where $$ I_{r}^{c}$$ is the complement set of $$ I_r$$, this is, the position with zero element in $$\mathbf{w}$$. This algorithm is called *pathSPCA*.

#### Sparse PCA Via Lasso Penalty: GPower

Journée et al. (2010) showed that the sparse PCA formulation based on maximizing the (scaled) standard deviation of the component scores using a lasso penalty,

9 $$\begin{aligned} \hat{\mathbf{w}}= \mathop {{\mathrm{argmax}}}\limits _{{\left\| \mathbf{w}\right\| =1 }}&\left\| \mathbf{Xw}\right\| -\lambda \left\| \mathbf{w}\right\| _{1}, \end{aligned}$$

is equivalent to solving initially:

10 $$\begin{aligned} \hat{\mathbf{z}}= \mathop {{\mathrm{argmax}}}\limits _{{\left\| \mathbf{z}\right\| \le 1 }}&\left\| S(\mathbf{X^{\top }}{} \mathbf{z}, \lambda ) \right\| ^{2}, \end{aligned}$$

where the soft-thresholding function $S(\mathbf{X^{\top }}{} \mathbf{z}, \lambda )$ is applied component wise. Once $\hat{\mathbf{z}}$ is obtained, define $\hat{\mathbf{w}} = S(\mathbf{X^{\top }}\hat{\mathbf{z}}, \lambda )/\left\| S(\mathbf{X^{\top }}\hat{\mathbf{z}}, \lambda ) \right\|$, which gives the sparsity pattern $S(\mathbf{X^{\top }}\hat{\mathbf{z}}, \lambda )$ for $\mathbf{w}$. Then, the component weights are obtained via the ordinary PCA (problem (4)) by removing the corresponding zero variables from the original data set $\mathbf{X}$. Note that the problem of solving for the *J*-dimensional vector $\hat{\mathbf{w}}$ is reformulated in terms of solving for a *I*-dimensional vector $\mathbf {z}$. In the high-dimensional setting, this avoids to search in a large space. A gradient scheme is used to solve problem (10). Additionally to the problem (9), Journée et al. (2010) also considered the problem of maximizing the variance subject to a cardinality penalty.

### Sparse PCA: Summary

PCA can be formulated as different optimization problems whose solutions happen to be equivalent (see page 7). However, when having sparsity constraints in the formulation, neither the SVD of the data set nor EVD of the covariance matrix is the solution of the sparse PCA problem. Given the lack of awareness of the different formulations and goals of PCA, it is not clear whatsoever when to use which method. In this section, we have discussed several methods for sparse PCA that all share the principle of Ockham’s razor to represent the data in a reliable though simple way. Table 1 summarizes the described methods: each of them imposes sparsity either on the component loadings or on the component weights. The last column of Table 1, “Algorithm”, indicates whether components are extracted one by one (deflation approach) or all together (block approach).

To impose sparsity, PCA methods rely on one of three popular techniques: rotation, the addition of a penalty, or a constraint (usually $l_0$ or $l_1$^7^). Many of the sparse PCA formulations are complex to solve, and a considerable amount of work is of an algorithmic nature; proposed algorithms are often subject to local optima and without guaranteed convergence. Moreover, some of the procedures also fail in terms of memory or are very slow to compute. Such algorithmic issues are not the focus here, yet they may affect the numerical performance of the methods.

**Table 1:** Summary of methods for sparse PCA.

Method	Estimated	Objective	Sparsity	Algorithm
VARIMAX	$\mathbf{P}$	Rotation	Threshold	Block
SIMPLIMAX	$\mathbf{P}$	Rotation	Threshold	Block
sPCA-rSVD	$\mathbf{P}$	low-rank	$l_1$	Deflating
SPCA	$\mathbf{W}$	Max. variance	$l_1$ and $l_2$	Block
pathSPCA	$\mathbf{W}$	Max. variance	$l_0$	Deflating
GPower	$\mathbf{W}$	Max. variance	$l_1$	Deflating

## Simulation Study

A crucial question that we want to address using simulated data is when to use which sparse PCA method. As discussed throughout the paper, choosing the proper approach depends on the assumed model (sparse component loadings, sparse component weights, or both) and performance of the method concerning various criteria. Here, we will use four measures to assess the performance of the six sparse PCA methods discussed in Sect. 2.

### Design

An essential factor in any simulation is the assumed data-generating model. Most of the reported simulation studies for sparse PCA are based on the spiked covariance model for which data follow a multivariate distribution with zero mean, variance $(\varvec{\Omega } =\mathbf {VDV}^{T})$, with sparse leading eigenvectors $\mathbf{V}_K$, and the *K* largest eigenvalues much larger than the remaining ones. Papers using this model include Zou et al. (2006), Shen and Huang (2008), Johnstone and Lu (2009). Another model that has been considered is the sparse standard factor model that relies on Eq. (1), that is, $\mathbf{X}=\mathbf{TP}^{\top }+\mathbf{E}$ with $\mathbf{P}$ sparse, and noise $\mathbf{E}$ independent of the components scores $\mathbf{T}$; see Adachi and Trendafilov (2016) for an example of a simulation study using this model. Also, more relaxed versions have been considered under the same name.^8^ Here, we will rely on three versions of the ‘factor model’ set up such that they correspond to the data model structure assumed by the sparse PCA methods considered in this study. First, consider

11 $$\begin{aligned} \mathbf{X}=\mathbf{TP}^{\top }+\mathbf{E} \end{aligned}$$

with $\mathbf{P}$ sparse and $\mathbf{T}^{\top }{} \mathbf{T}=\mathbf{I}$; note that model in Eq. (11) corresponds to the structure imposed by Adachi and Trendafilov (2016). Second, considering the component scores explicitly as a function of the weights,

12 $$\begin{aligned} \mathbf{X}=\mathbf{XWP}^{\top }+\mathbf{E} \end{aligned}$$

with $\mathbf{W}$ sparse and, third, the same model in Eq. (12) but, with $\mathbf{P}$ and $\mathbf{W}$ being sparse simultaneously.

For generating the synthetic data sets, besides the data-generating model, we also considered the following factors and levels: sample size with levels $I= 100, 500$, number of variables with levels $J = 10, 100,1000$, number of components with levels $K = 2,3$, percentage of variance accounted for the data set with levels $\text {VAF} = 80\%,95\%,100\%$, and proportion of sparsity with levels $\text {PS}=0.0, 0.5, 0.8$ or $\text {PS} = 0.7,0.8, 0.9$ when data are generated with component loadings and component weights being equal, sparse, and orthogonal. These higher levels of sparsity allow avoiding overlap of the nonzero values making it possible to have sparse structures that are orthogonal. For each of the three types of models, a fully crossed design was used, resulting in $2 \times 3 \times 2 \times 3 \times 3= 108$ conditions. For each condition, 100 data sets were generated, ending up with a total of 10, 800 data sets in each of the three data generating regimes. The data generation design is summarized in Table 2.

**Table 2:** Simulation design factors and their levels.

Model	sparse	*I*	*J*	*K*	*VAF*	*PS*	Repetions
$\mathbf{X}=\mathbf{TP}^{\top }+\mathbf{E}$	$\mathbf{P}$	100, 500	10, 100, 1000	2, 3	80%, 95%, 100%	0.0, 0.5, 0.8	100
$\mathbf{X}=\mathbf{XWP}^{\top }+\mathbf{E}$	$\mathbf{W}$	100, 500	10, 100, 1000	2, 3	80%, 95%, 100%	0.0, 0.5, 0.8	100
$\mathbf{X}=\mathbf{XWP}^{\top }+\mathbf{E}$	$\mathbf{P}$ and $\mathbf{W}$	100, 500	10, 100, 1000	2, 3	80%, 95%, 100%	0.7, 0.8, 0.9	100

*I* sample size, *J* No. of variables, *K* N. of components, *VAF* variance accounted, *PS* proportion of sparsity

Data were generated using one of three algorithms: Algorithm 1 is used for generating data with a sparse component loadings structure, Algorithm 2 generates data with a sparse component weights structure, and Algorithm 3 generates data with, orthogonal and equal sparse component loadings and weights. Every algorithm begins with a rank-*K* decomposition obtained from the truncated SVD decomposition of data generated from a multivariate normal distribution. Algorithm 1 then imposes sparsity on the component loadings $\mathbf{P = V}{} \mathbf{D}$ and has orthogonal component scores $\mathbf{T=U}$; Algorithm 2 imposes sparsity on the component weights $\mathbf{W=V}$. For Algorithm 3 there are two scenarios: (1) For the model that assumes $\mathbf{P}$ sparse, $\mathbf{W = V}{} \mathbf{D}^{-1}$, and (2) for the models that assumes $\mathbf{W}$ sparse, $\mathbf{P=V}$. Additionally, every algorithm considers additive noise $\mathbf{E}$ distributed according to a multivariate normal distribution with mean $\mathbf{0}$, and variance proportional to the identity matrix, such that the final data set has the desired VAF. This error structure has been also considered in leading sparse PCA papers (e.g., Johnstone and Lu 2009; Shen and Huang 2008; Zou et al. 2006), while Van Deun et al. (2019) considers generalizations of sparse PCA to data with non-additive noise.
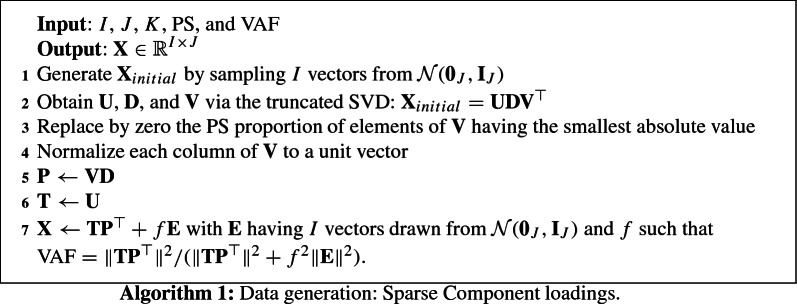

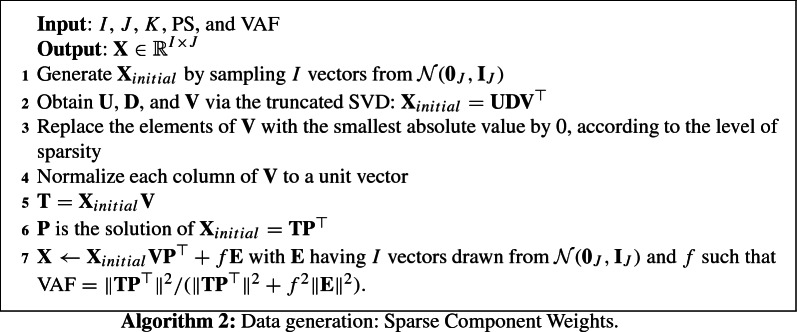

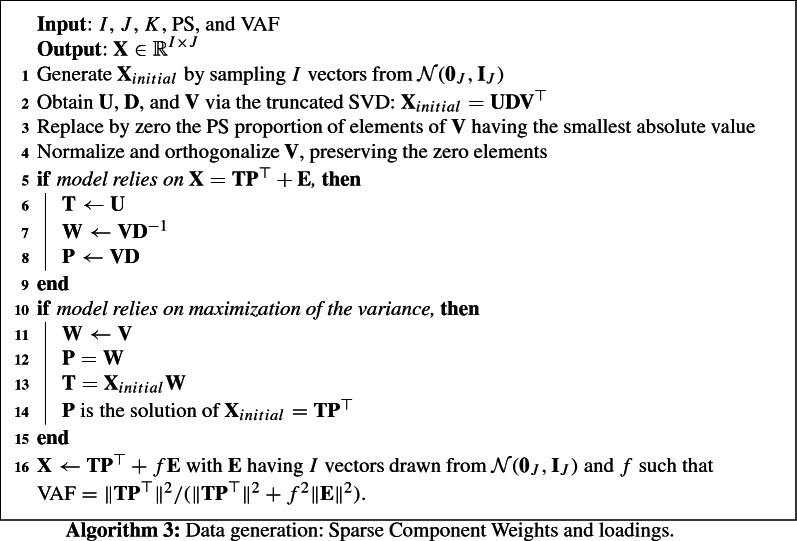


Each data set was analyzed using the six sparse PCA methods previously discussed: PCA with simple thresholding of the rotated loadings using either Varimax or Simplimax rotation, sPCA-rSVD, SPCA, pathSPCA, and GPower. Also, the performance of each method on each data set was assessed using the following performance measures: the squared relative error (SRE) of the model parameters, the misidentification rate (MR) of zero versus the nonzero status of the sparse coefficients, the percentage of explained variance (PEV), and the cosine similarity (also known as Tucker’s coefficient of congruence). The performance measures are defined as follows.

- The SRE is used to assess how well each method estimates the model component scores, component loadings, and/or component weights. For a matrix $\mathbf{A}$, the SRE is defined by
  $$\begin{aligned} \text {SRE}(\mathbf{A}) = \frac{ \left\| {{\widehat{\mathbf{A}}}-\mathbf{A}}\right\| _{F}^{2}}{\left\| {\mathbf{A}}\right\| _{F}^{2} }, \end{aligned}$$
  with $\widehat{\mathbf{A}}$ representing the estimated matrix. Values close to zero indicate good recovery of the original model matrix by the method, while values close to or higher than one indicate bad recovery. The SRE is calculated for the component scores $\mathbf{T}$, component loadings $\mathbf{P}$, and component weights $\mathbf{W}$. The cosine similarity (or Tucker congruence) between matrices $\mathbf{A}$ and $\mathbf{B}$ with dimension $I\times K$ is defined as
  13 $$\begin{aligned} CosSim(\mathbf {A,B})= \frac{1}{K}\sum _{k=1}^{K} \frac{ \mathbf {a}_k^{\top }{} \mathbf {b}_k }{\left\| \mathbf {a}_k \right\| \left\| \mathbf {b}_k \right\| } \end{aligned}$$
  with $\mathbf{a}_k$ and $\mathbf{b}_k$ the *k*-th column of matrix $\mathbf{A}$ and $\mathbf{B}$, respectively. This value is calculated between the estimated component loadings and the population component weights $CosSim(\widehat{\mathbf{P}},\mathbf {W})$, the estimated component weights and the population component loadings $CosSim({\widehat{\mathbf{W}}},\mathbf {P})$, and the estimated and population component scores $CosSim({\widehat{\mathbf{T}}},\mathbf {T})$. The *CosSim* is only calculated for the simulation settings representing a mismatch between the sparse constraints imposed by the data generating model and those imposed by the method.
- The misidentification rate assesses how badly each model captures the sparse structure of the data set. MR is defined as the percentage of zero values that are not recovered, that is,  MR is a value in the interval [0, 1]. When $\text {MR}=0$, all zeros in the generated model structure have been estimated as a zero by the sparse PCA method, while $\text {MR}=1$ means that none of the zeros in the model structure has been estimated as a zero by the method. Hence, methods set up to identify the underlying sparse structure should have $\text {MR}$ values close to zero. Note that in simulation conditions with the proportion of sparsity set to zero, the MR is not calculated.
- The percentage of explained variance was implemented to assess how well the sparse component solution explains the variance in the generated data. PEV is defined as
  $$\begin{aligned} \text {PEV} =1- \frac{ \left\| {{\widehat{\mathbf{X}}}-\mathbf{X}}\right\| _{F}^{2}}{\left\| {\mathbf{X}}\right\| _{F}^{2} }. \end{aligned}$$
  where ${\widehat{\mathbf{X}}}$ represents the recovered data set and it is defined as ${\widehat{\mathbf{X}}} = {\widehat{\mathbf{T}}\widehat{\mathbf {P}}^{\top }}$. PEV is a value in the interval [0, 1] and is desired to be close to the variance accounted by the generated data (VAF); a PEV value greater than VAF means that the model extracts some of the residual variation (i.e., the noise), which is a sign of overfitting.

Note that—except for PEV—all performance measures are sensitive to order permutations and changing of the sign of the component scores, loadings, or weights. However, the methods considered here have sign invariance, and some of them also have permutational invariance. Therefore, to make our measurement robust, we considered all possible permutations of the component loadings/weights—including changes of their sign—and calculated all measurements with the combination that produces the minimum SRE (or *CosSim* when is used).

### Results

#### Overview

**Fig. 1:**
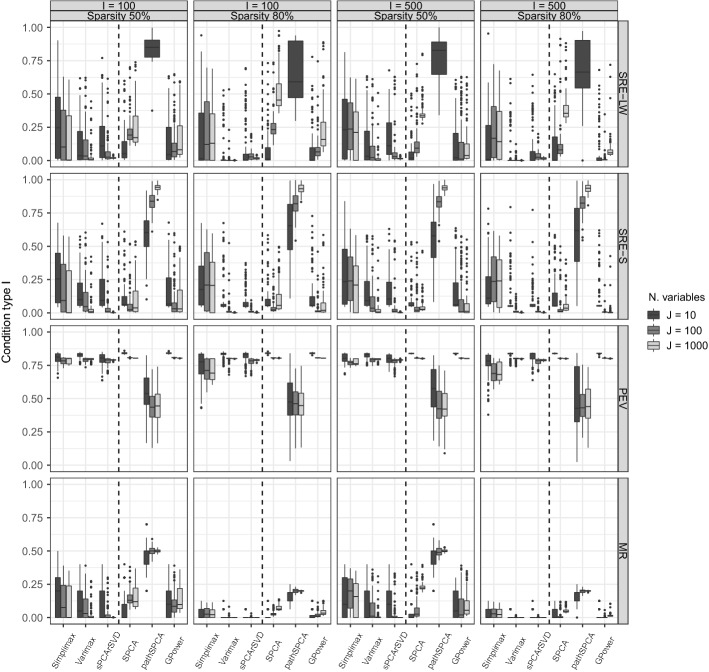
Matching sparsity: Boxplots of the performance measures in conditions with 80% of variance accounted by the model in the data and two components. Within each panel, a dashed line divides the boxplots for sparse loadings methods (at the left side of the dashed line) from those for sparse weights methods. The top row summarizes the squared relative error (SRE-LW) for the loadings (at the left of the dashed line) and weights (at the right of the dashed line), the second row the SRE-S for the component scores, the third row (PEV) the proportion of variance in the data explained by the estimated model, and the bottom row the misidentification rate (MR).

**Fig. 2:**
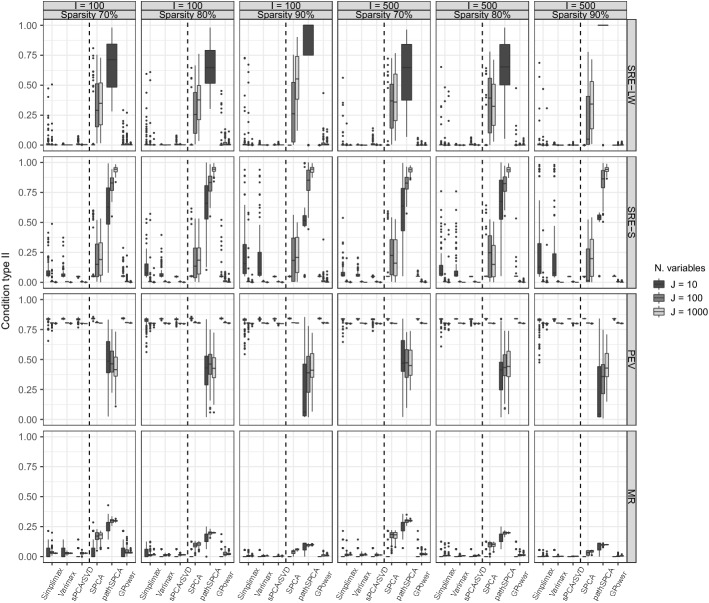
Double sparsity: Boxplots of the performance measures in conditions with 80% of variance accounted by the model in the data and two components. Within each panel, a dashed line divides the boxplots for sparse loadings methods (at the left side of the dashed line) from those for sparse weights methods. The top row summarizes the squared relative error (SRE-LW) for the loadings (at the left of the dashed line) and weights (at the right of the dashed line), the second row the SRE-S for the component scores, the third row (PEV) the proportion of variance in the data explained by the estimated model, and the bottom row the misidentification rate (MR).

**Fig. 3:**
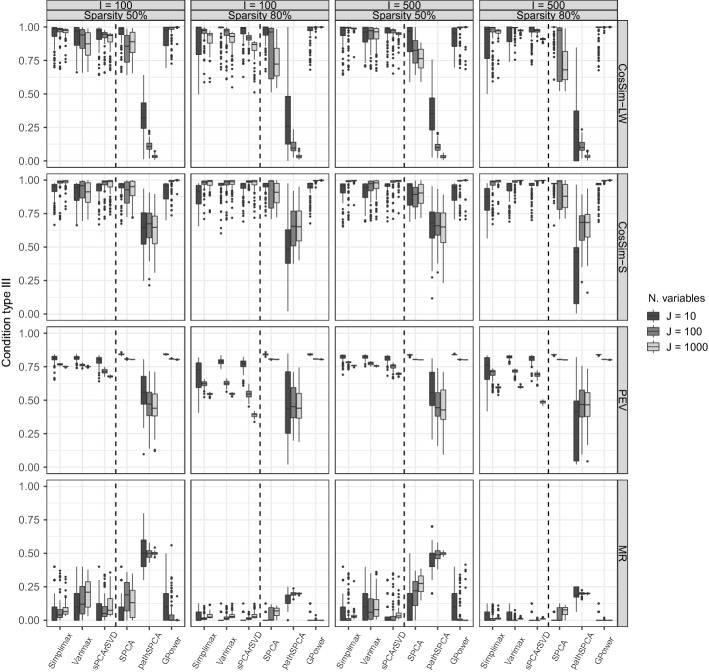
Mismatching sparsity: boxplots of the performance measures in conditions with 80% of variance accounted by the model in the data and two components. Within each panel, a dashed line divides the boxplots for sparse loadings methods (at the left side of the dashed line) from those for sparse weights methods. The top row summarizes the squared relative error (SRE-LW) for the loadings (at the left of the dashed line) and weights (at the right of the dashed line), the second row the SRE-S for the component scores, the third row (PEV) the proportion of variance in the data explained by the estimated model, and the bottom row the misidentification rate (MR).

We present the results for three different types of conditions. In condition type I, the sparse structure of the generated data matches the sparse structure of the methods. In condition type II, the data have been generated with more constraints than those set by the methods. Finally, in condition type III, we assume a mismatch between generated and estimated sparse structures (that is, analyzing data generated with sparse loadings using a method that yields sparse weights and vice versa, see Table 3). In Figs. 1, 2, and 3 , we report results for the settings that include two components, a PS equal to 50% and 80% for condition types I and III, and VAF equal to 80%. Each panel contains a boxplot of a performance measure. Within each panel, a dashed line divides the boxplots for sparse loadings methods (at the left side of the dashed line) from those for sparse weights methods. For condition type II, the settings with two components scores and VAF equal to 80% were included.^9^ All analyses were performed using the actual values of the number of components and the sparsity level available in the simulation setting. Therefore, differences in performance are not the result of an improper tuning of the meta-parameters by the methods.

**Table 3:** Simulation description summary.

Condition	Sparse structure	Algorithm	Measurements		
Type I	$\mathbf{P}$	Alg-I	SRE	MR	PEV
	$\mathbf{W}$	Alg-II	SRE	MR	PEV
Type II	$\mathbf{P}$ and $\mathbf{W}$	Alg-III	SRE	MR	PEV
	$\mathbf{P}$ and $\mathbf{W}$	Alg-III	SRE	MR	PEV
Type III	$\mathbf{W}$	Alg-II	CosSim	MR	PEV
	$\mathbf{P}$	Alg-I	CosSim	MR	PEV

#### Condition Type I: Matching Sparsity

The first type of conditions that we discuss are those with data generated using the same model structures as the corresponding methods. Therefore, data generated by Algorithm 1 were analyzed with thresholding of rotated loadings and sPCA-rSVD, while data generated by Algorithm 2 were analyzed with SPCA, pahSPCA, and GPower. Figure 1 shows the results of the different performance measures for the simulation setting with two components and VAF equal to 80%. It can be observed that among the methods with sparse loadings, both thresholded Varimax and sPCA-rSVD perform reasonably well on all performance measures and in all settings. Thresholded Simplimax, on the other hand, only performs well with respect to explaining the variance. Comparing Varimax with sPCA-rSVD, we found that sPCA-rSVD has the lowest MR in all conditions and has a better recovery of the loadings and component scores in situations with many variables ($J>10$). We found a strong effect of the level of sparsity on the MR. MR is lower when the PS is higher: This is mainly an artefact as the maximal MR is $1-.6/.8 = 0.25$ when the sparsity is 80% and 1 when it is 50%. For Varimax and sPCA-rSVD (and in some conditions also for Simplimax), some effect of the number of variables can be observed: Better results were obtained when the number of variables increases. This is contrary to expectations, given reported issues for high-dimensional data (see Sect. 1.2). However, as explained previously in Sect. 2, the estimation of the loadings with the sPCA-rSVD method boils down to univariate regressions.

Among the methods imposing sparsity on the weights, GPower shows the best performance in general. For the SRE on the component weights and component scores (first and second row), it always had the lowest values when the proportion of sparsity was 80%. For different parameter settings, GPower and SPCA presented similar results. Related to the PEV and MR, GPower and SPCA showed favorable performance, although GPower obtained the best performance on the latter. Both for SPCA and GPower, it holds that their SRE performance decreased with an increasing number of variables; the estimation problem, with sparse component weights, suffers from the high-dimensionality as the estimation of the weights streamlines to a high-dimensional regression problem. Finally, pathSPCA had the worst performance on every measure. For the MR, pathSPCA obtained values close to the maximum possible, and the SRE were always close to or greater than 1.

#### Condition Type II: Double Sparsity

In condition type II, the data were generated with the component loadings and component weights simultaneously sparse, relying on Algorithm 3. Figure 2 shows the results for the performance measures in the conditions with two components and VAF equal to 80%. We found that sPCA-rSVD and GPower maintained good performance and showed the best performance for sparse loadings and sparse weights methods, respectively. Both rotation techniques and sPCA-rSVD performed better in general with a reduction of the SRE of the component loadings and scores, a reduction of the MR, and a slight increment of the PEV. The performance of SPCA is much worse in the settings with 100 and 1, 000 variables for all measures but the PEV, which remains around 80%. PathSPCA still performs badly, especially with respect to MR, where it almost attains the maximum possible value.

Besides comparisons within methods imposing sparsity on $\mathbf {P}$ and within methods imposing sparsity on $\mathbf {W}$, comparisons between the two purposes can also be made $(\mathbf {P} vs \mathbf {W})$. In condition type I and II, sPCA-rSVD outperformed GPower on all measures but PEV, where they showed similar performance. This indicates that methods for sparse component loadings recover better the sparse component loading structure than that methods for sparse component weights recover the sparse component weight structure. The comparison also indicates that sparse component weights methods have higher PEV.

#### Condition Type III: Mismatching Sparsity

In condition type III, the sparse structures were mismatched between generated and estimated structures, that is, data generated with sparse component weights were analyzed with sparse loadings methods while data with sparse component loadings were analyzed with methods for sparse weights. This implies that sparse loadings methods were assessed using data generated with Algorithm 2, and sparse weights methods are assessed using data generated with Algorithm 1. Additionally, the similarity measure described in Eq. (13) was used to assess the recovery of the component loadings/weights and scores instead of SRE.

Figure 3 summarizes the results for the setting with two components and VAF equal to 80%. Note that for the sparse loadings methods, the recovery of the component weights is calculated (and thus not of the component loadings), while for sparse weights methods the recovery of the component loadings is calculated. All methods for sparse loadings—thus imposing sparse component loadings—recover the *component weights* and component scores well; Simplimax even obtains better results than Varimax in the conditions with 50% of sparsity and in some conditions also than sPCA-rSVD. Compared to condition types I and II, when 80% sparsity is imposed and $J>I$ the PEV drops. This can be understood by the fact that data were generated with sparse component weights while they were estimated with sparse component loadings, the latter having a more direct impact on the recovered data ${\hat{x}}_{ij}$ than the former.

Methods for sparse weights show the same pattern of results as in condition type I and notably maintain the same PEV as in condition types I and II. GPower outperformed SPCA in most of the settings and measures, although the latter still shows reasonably good results except with respect to MR in the high-dimensional settings. Compared to condition type I, GPower also outperformed SPCA on the MR in conditions with 50% of sparsity; its performance improved in this condition with mismatched sparsity. PathSPCA performed badly on every measure. Additionally, GPower outperformed sPCA-rSVD on all measures and in almost all conditions except for those with $J=10$. Taken together, these results suggest that an underlying sparse component loading structure can be recovered better by a sparse component weight method and with higher PEV than vice versa.

**Fig. 4:**
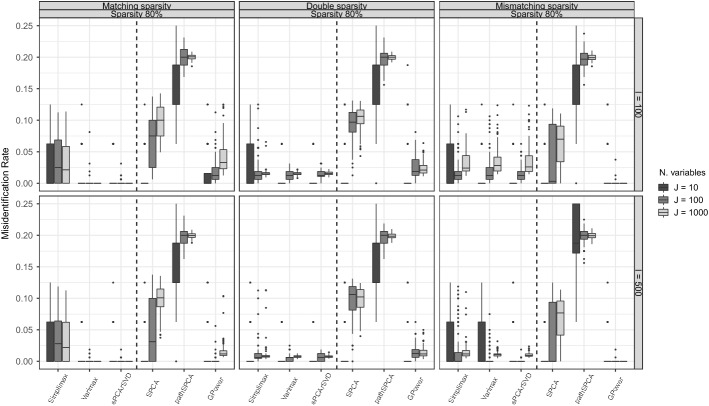
Misidentification rate (MR): boxplots of the MR in conditions with 80% of variance accounted by the model in the data, a proportion of sparsity of 0.8, and two components. Within each panel, a dashed line is used to divide the boxplots for sparse loadings methods (at the left side of the dashed line) from those for sparse weights methods.

**Fig. 5:**
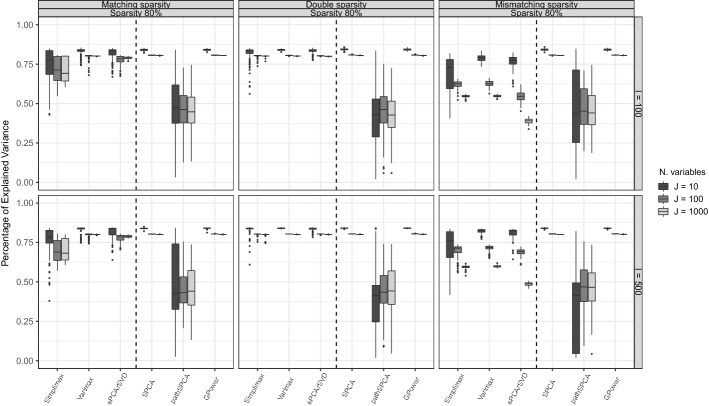
Percentage of explained variance (PEV): boxplots of the PEV in conditions with 80% of variance accounted by the model in the data, a proportion of sparsity of 0.8, and two components. Within each panel, a dashed line is used to divide the boxplots for sparse loadings methods (at the left side of the dashed line) from those for sparse weights methods.

We used Figs. 4 and 5 to summarize the MR and PEV of the three condition types. First we discuss MR. The robustness of the methods in capturing the sparse structure under varying data generation schemes can be observed in Fig. 4. We can see, for example, that Simplimax showed its best MR in the conditions where sparseness is imposed on the component weights (condition types II and III). On the other hand, Varimax and sPCA-rSVD showed their best results in condition type I. SPCA presented good results only when $I=10$ for the three condition types. GPower, although being a method that imposes sparseness on the weights, has a better recovery of the sparse structure when data are generated with sparse loadings (condition types II and III). Second, regarding the PEV (see Fig. 5), GPower and SPCA showed the best PEV under each condition type, and methods for sparse loadings only have a comparable PEV when data were generated with sparseness both on loadings and weights (condition type II). On both measures, MR and PEV, pathSPCA consistently showed poor performance across every condition type. Additionally, comparing the MR of GPower (sPCA-rSVD) in condition type I with sPCA-rSVD (GPower) performance in condition type III, we see that the sparse loading structure of sPCA-rSVD does a better job in finding the sparse structure of component weights for data generated with a sparse component weight structure. GPower, however, is not better in finding the underlying sparse loading structure than sPCA-rSVD.

The different results in condition types I and II that we observe in Fig. 4 further support the hypothesis that sparse component loadings and sparse component weights should be treated differently. If sparse component loadings and sparse component weights were the same, we would have observed the same results in conditions type I and III, which is not the case. In condition type II, it is assumed that both component loadings and weights have the same sparse structure, and methods for sparse loadings showed a better performance recovering the sparse structure in the data sets.

### Summary

Here we focus on two essential aims of a sparse PCA analysis, namely recovering the sparseness structure (which variables are associated with the components and which ones not) and explaining maximal variance in a parsimonious way. (This is using components that are a linear combination of a few variables only.) When recovery of the sparseness structure is the aim, a sparse loading approach (preferably sPCA-rSVD) should be used unless the data have an underlying sparse weight structure. (In the latter case, the GPower approach with sparse weights should be used). When summarizing the variables with a few derived variables that explain maximal variance and are based on a linear combination of a few variables only is the goal, a sparse weight approach should be used, preferably GPower.

Although the present results convincingly favor sPCA-rSVD and GPower, we should acknowledge that we unrealistically used knowledge about the number of components and the level of sparseness to implement the methodologies. These factors’ actual values are only available in simulation studies and not when using empirical data sets. Then, parameters such as the proportion of sparsity and the number of components require additional techniques to select them. Those techniques are out of the scope of this study. The following section illustrates the implementation of sparse PCA methodologies using empirical data sets.

## Empirical Applications

In this section, we use two empirical data sets to illustrate the application of sparse PCA in practice. We used a highly structured data set with variables designed to measure one of five underlying psychological constructs. Here the aim of the sparse PCA analysis is to reveal the sparse structure that underlies the data: each variable is expected to be associated to one component only. A second data set was selected to show the use of sparse PCA as a summarization tool in the high-dimensional setting. For this purpose, we analyze a ultra-high-dimensional genetic data set with the aim of finding a limited set of genes that allow to classify subjects into one of three groups (two autism-related groups and a control group).

An important issue that needs to be addressed for these empirical applications, and that was not addressed in the simulation study, is the choice of the number of components and the level of sparsity. For the number of components, we rely on the literature and substantive arguments made therein. For the proportion of sparsity, we rely on a data driven method, namely the *Index of sparseness (IS)* introduced by Trendafilov (2014), that was shown to outperform other methods such as cross-validation and the BIC in estimating the true proportion of sparsity (Gu et al. 2019). The *IS* is defined as

$$\begin{aligned} {\text {IS}} = {\text {PEV}}_\mathrm{sparse}\times {\text {PEV}}_\mathrm{pca}\times {\text {PS}} \end{aligned}$$

with ${\text {PEV}}_\mathrm{sparse}$, ${\text {PEV}}_\mathrm{pca}$, and PS denoting the PEV using a sparse method, PEV using ordinary PCA, and the proportion of sparsity (loadings or weights), respectively. The IS value increases with the goodness-of-fit ${\text {PEV}}_\mathrm{sparse}$, the higher adjusted variance ${\text {PEV}}_\mathrm{pca}$, and the sparseness: the level of sparsity is determined by maximizing IS.

### Big Five Data

**Fig. 6:**
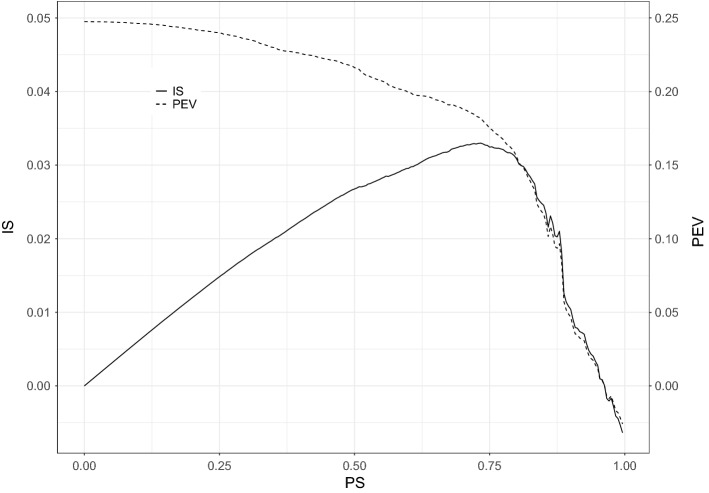
*Index of sparseness*(IS) and percentage of explained variance (PEV) against the proportion of sparsity (PS).

We used data on the Big Five personality dimensions publicly available from the R-package *qgraph* (Epskamp et al. 2012), henceforth called Big Five data. The data set contains the scores of 500 individuals on the NEO-PI-R questionnaire (McCrae and Costa 1999) consisting of five sets of 48 items (i.e., 240 items in total), each set measuring one of the Big Five personality traits (Neuroticism, Extraversion, Openness to Experience, Agreeableness, and Conscientiousness) (Dolan et al. 2009). For this kind of data, interest is usually in the correlation patterns in the data (component loadings); therefore, each variable was mean-centered and scaled to unit variance. Following the design of the questionnaire, we chose $K=5$ five components. Ordinary PCA explained $24\%$ of the total variance; this is the maximal amount of variance that can be explained with 5 components. We will analyze these data with six sparse PCA methods. Yet, before doing so, we first need to tune the level of sparseness. As sPCA-rSVD showed the best performance in the simulation study, we use this method in combination with IS to determine the level of sparseness. Figure 6 shows the values for the IS and PEV as a function of the proportion of sparsity for sPCA-rSVD, calculated as the proportion of the $5 \times 240$ loadings that are zero. The maximum IS for sPCA-rSVD is attained at a sparsity proportion of 0.73 having $18\%$ explained variance. This proportion of sparsity corresponds to a sparse model having only 64 nonzero out of 240 loadings for each component; this is reasonably close to the 48 nonzero loadings that may be expected on the basis of the design of the questionnaire.

**Fig. 7:**
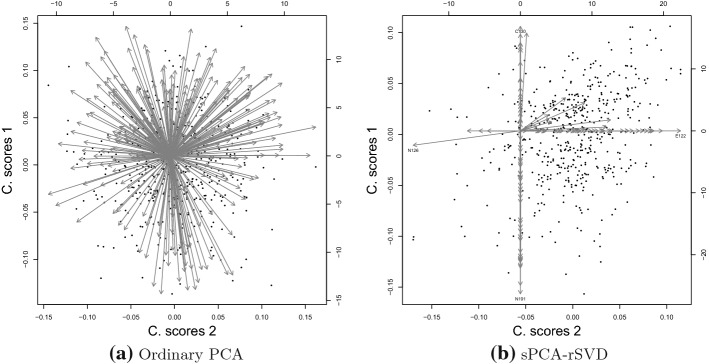
Biplot: the dots in each subplot represent the component scores, the arrows the component loadings.

The biplot representation of the first two components after running PCA and SPCA-rSVD is shown in Fig. 7. Each variable is represented by an oriented vector and each subject by a dot. Figure 7a depicts the first two PCA components. Each item loads on both components, and the solution is hard to interpret; sparseness has been introduced to improve interpretability. The biplot representation of the two first sPCA-rSVD components is shown in Fig. 7b. Most of the items load just on one component; this makes interpretation of the components easy.

Table 4 presents a summary of the number of items in each set that have a nonzero loading for the five components. Using sPCA-rSVD, except for the fourth component, most nonzero loadings belong to one particular item set. For instance, from the 64 items that load on component 1, 34 belong to Neuroticism and 17 to Extraversion; on the other hand, items having a nonzero loading on component 2, mainly belong to Agreeableness (29 items), and Extraversion (19 items). Hence, the components are strongly associated with one specific trait; this is especially true for the third component (mainly Conscientiousness items) and fifth component (mostly Openness items). On the fourth component, relatively many items from both Extraversion and Agreeableness load. The prior expectation may be that the items of one set load only on one particular component and thus it invalidates the sPCA-rSVD method. Yet, many studies have shown the type of pattern found here, for example, high cross-loadings for Extraversion and Agreeableness after Procrustes rotation to the predefined structure (McCrae et al. 2005).

To illustrate the comparative performance on the same empirical data, we implemented the other methods using the Big Five data set with the total number of nonzero coefficients fixed to the one found for sPCA-rSVD. As can be seen from Table 4, the Varimax results largely reflect the design underlying the questionnaire with items designed to measure a particular trait loading only on one particular component. Simplimax, on the other hand, does not recover the underlying structure; it has no component that is clearly dominated by the extraversion items, and the conscientiousness trait does not show up as a single component but rather as two (components 2 and 3). Using methods with sparse weights, the zero/nonzero pattern of the SPCA weights is very similar to the pattern of the Simplimax loadings. However, SPCA explains only 13% of the variance. PathSPCA showed no particular structure, each component is a weighted combination of variables related to all traits, and these components explain only 9% of the variance. Finally, by using GPower, 22% of the variance can be explained. However, the summary representations by the GPower components do not include the variables related to the Neuroticism; this trait practically disappeared. Only two and one variable of the Neuroticism set of items have a nonzero weight for component 1 and 2, respectively. Additionally, items designed to measure the Openness trait underlie three of the five components (namely, components 2, 3, and 5).

Overall, the results presented in Table 4 highlight the importance of taking the purpose of analysis into account when choosing the sparse PCA method. We observe that methods imposing sparseness on the loadings are more suitable for the purpose of exploratory data analysis than methods imposing sparseness on the component weights. The sparsity pattern of the sPCA-rSVD and Varimax loadings reflected the questionnaire design underlying the data best even though the latter showed poor performance on every performance measure in the simulation study. On the other hand, GPower explained the most variance but could not recover the personality traits from the data. Finally, in line with the simulation study, pathSPCA failed to explain a reasonable amount of variance and to recover the underlying traits.

**Table 4:** Sparse loading and weights composition by trait (OCEAN).

	sPCArSVD					Varimax					Simplimax				
	$\mathbf{p}_1$	$\mathbf{p}_2$	$\mathbf{p}_3$	$\mathbf{p}_4$	$\mathbf{p}_5$	$\mathbf{p}_1$	$\mathbf{p}_2$	$\mathbf{p}_3$	$\mathbf{p}_4$	$\mathbf{p}_5$	$\mathbf{p}_1$	$\mathbf{p}_2$	$\mathbf{p}_3$	$\mathbf{p}_4$	$\mathbf{p}_5$
Openness	0	9	1	4	41	1	0	8	5	42	0	17	9	4	30
Concientiousness	9	3	11	43	2	7	7	3	44	4	15	0	23	31	7
Extraversion	17	19	21	6	9	16	15	30	5	7	15	10	6	7	11
Agreeableness	4	29	23	2	5	3	33	16	4	4	6	33	13	14	5
Neuroticism	34	4	8	9	7	37	9	7	6	7	28	4	13	8	11
Total nonzero	64	64	64	64	64	64	64	64	64	64	64	64	64	64	64
	SPCA					pathSPCA					Gpower				
	$\mathbf{w}_1$	$\mathbf{w}_2$	$\mathbf{w}_3$	$\mathbf{w}_4$	$\mathbf{w}_5$	$\mathbf{w}_1$	$\mathbf{w}_2$	$\mathbf{w}_3$	$\mathbf{w}_4$	$\mathbf{w}_5$	$\mathbf{w}_1$	$\mathbf{w}_2$	$\mathbf{w}_3$	$\mathbf{w}_4$	$\mathbf{w}_5$
Openness	0	17	4	13	25	16	12	14	12	10	27	4	12	41	33
Concientiousness	15	0	26	24	8	15	15	11	10	13	11	3	42	11	15
Extraversion	15	10	15	6	16	16	10	14	14	10	3	34	5	10	12
Agreeableness	6	27	13	10	3	15	9	11	17	12	39	4	1	5	5
Neuroticism	28	10	6	11	12	17	10	12	9	16	1	2	0	0	0
Total nonzero	64	64	64	64	64	79	56	62	62	61	81	47	60	67	65

Each column represents the number of items in each loading/weight that have a nonzero value in each trait. The components were ordered such that the number of nonzero loading/weights on the diagonal is maximized

### Gene Expression Data

To illustrate sparse PCA used as a summarization tool, we rely on publicly available gene expression data comparing 14 male control subjects to 13 male autistic subjects^10^. The autism subjects were further subdivided in two groups: a group of six with autism caused by a fragile X mutation (*FMR1-FM*) and a group of seven with autism caused by a 15q11–q13 duplication (*dup15q*). For each subject the transcription rates of 43, 893 probes, corresponding to 18, 498 unique genes, were obtained; hence the number of variables is much larger than the number of observations, with known numerical issues for generalized linear models (Hastie et al. 2001). Often the approach followed to account for such high-dimensionality is to first reduce the large set of variables to a few components. Because it showed the best performance in the simulation study, we will use GPower method to select the relevant genes that summarize the component scores.

**Fig. 8:**
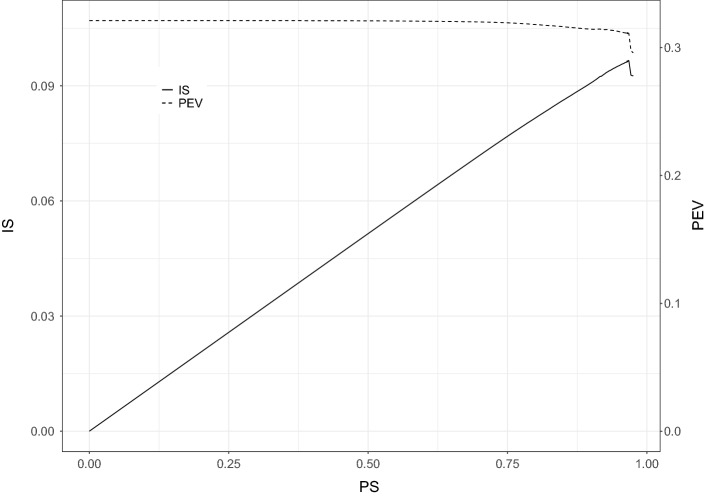
*Index of sparseness* and percentage of explained variance against the proportion of sparsity when applying GPower to the gene expression data set.

Prior to analyzing the data, we centered and scaled them to unit variance; in this way we focus on the correlation between the expression values. Following the original publication, we select $K=3$ three components ( Nishimura et al. 2007). Figure 8 shows the *IS* and PEV as a function of the proportion of sparsity. The maximal PEV with three components, obtained with ordinary PCA, accounts for $32\%$ of the total variance. The maximum value of *IS* is reached at a proportion of sparsity of 0.97 with a PEV of $31\%$. This corresponds to 3% or 4, 323 nonzero component weights, spread over 4, 323 different variables each having exactly one nonzero weight. Therefore, we found an efficient reduction of the high-dimensional data to just three derived variables (the component score vectors) using approximately $10\%$ of the original variables while losing only $1\%$ of the variance accounted compared to when all variables are used in constructing the components via ordinary PCA.

When using the other sparse PCA methods, only sPCA-rSVD can handle the dimension of the data set computationally. However, if sPCA-rSVD had been used as a summarization tool with the same optimal proportion of sparseness found for GPower (PS=0.97), virtually 0% of the variance would have been explained, evidencing that methods imposing sparsity in the weights are more suitable for summarization purpose.

**Fig. 9:**
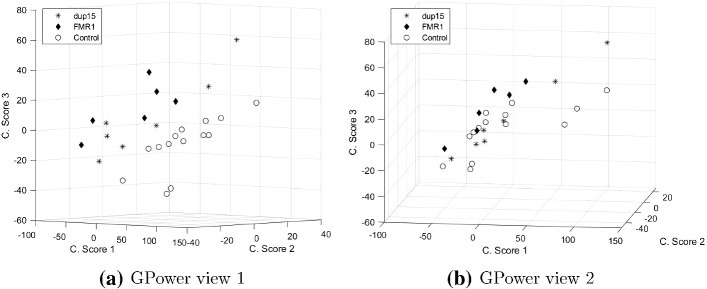
Scatter plot of component scores.

Figure 9 shows the scatter plot of the three component scores. From Fig. 9a, we observe that the first component separates the individuals with autism from the control group; this could be expected as the largest source of variation in the data is the distinction between control and autistic subjects. One may notice that Nishimura et al. (2007) constructed components scores using a subset of 293 probes with significant difference in expression between the three groups in an analysis of variance (ANOVA). In other words, the authors used an informed approach to select the relevant genes while sparse PCA methods (here GPower) do not rely on such external information; still, a separation between the two large groups can be observed from Fig. 9b.

## Concluding Remarks

As explained in this study, different PCA formulations give the same estimated scores and lead to estimates of the model coefficients that are the same or only differ up to scaling or rotation. Not surprisingly, little attention has been given to existing differences between the PCA methods, which is exemplified by the different meanings given to the term ‘loadings’ in the literature. Based on these different formulations of PCA, different methods for sparse PCA have been proposed where most of the attention has been given to the different ways of imposing sparsity and the numerical procedures used to solve the optimization problems. But, the sparse PCA methods are different on a more fundamental level and this is seldom discussed; the (implicitly) assumed data-generating model is often overlooked, while sparsity is imposed on different model structures (either the component weights or the component loadings). Also sparse PCA may serve different purposes in which some methods may be better than other ones. For instance, for exploratory data analysis, finding structure in the data and attaching meaning to the components is of primary importance. Then, good recovery of the relevant variables and the structure therein is required. For summarization, the primary focus is to find component scores that maximally account for the variance in the data. Here, the focus is on the proportion of explained variance and, sometimes, on recovering the component scores.

To offer users of sparse PCA guidance on which method to use and under what circumstances, in a simulation study, we compared six popular methods under three data-generating schemes and four performance measures. Assuming matching sparsity (e.g., generating data with a sparse loading model and estimating them back with a method for sparse loadings), sPCA-rSVD was the preferred method based on every performance criterion for sparse loadings methods, and GPower was the best method among the sparse weights methods. In psychology, a common practice is to threshold the loadings obtained after rotation to a simple structure. In our simulation study, thresholding sometimes gave good results but sometimes also produced much worse results than the sPCA-rSVD approach. Considering that the data generating model may be unknown and that there may be a mismatch in sparsity, sPCA-rSVD is overall the best method for recovering the relevant variables, and GPower performs best in terms of explained variance.

Finally, from a practical point of view, the availability of software is of utmost importance for the use of data analysis methods. Unfortunately, sPCA-rSVD and GPower have not been (yet) implemented in major software packages such as SPSS. GPower, to our knowledge, is currently only available in MATLAB. sPCA-rSVD with a cardinality constraint is available in the *ClusterSSCA* R-package (Yuan et al. 2019), while a penalized approach is part of the *RegularizedSCA* R-package (Gu and Van Deun 2019).

## Supplementary Information

Below is the link to the electronic supplementary material.

Supplementary material 1 (pdf 439 KB)

## Footnotes

1 The MATLAB and R codes used to perform simulation study and applications are available from https://github.com/RosemberGuerra/sparsePCA.

2 It can be shown that the element $p_{j,k}$ is the correlation between variable $\mathbf{x}_j$ and component scores $\mathbf{t}_k$.

3 Ning-min and Jing (2015), Trendafilov (2014), Zou and Xue (2018) give a wide list of more methods for both purposes.

4 Observe that from (1) it follows that $\mathbf{x}_j = \sum _k \mathbf{t}_k p_{j,k} + \mathbf{e}_j$ which is the linear regression equation with dependent variable $\mathbf{x}_j$ and predictor variables $\mathbf{t}_k$.

5 A non-singular matrix $\mathbf{Q}\in {\mathbb {R}}^{K\times K}$ is called oblique if $\mathbf{Q^\top Q}$ is a correlation matrix (Trendafilov 2014).

6 Referred as loadings in Zou et al. (2006).

7 Note that for $l_1$ it is possible to find a dual representation though this is not always the case for the $l_0$ pseudo-norm; see, e.g., Bertsimas et al. (2016).

8 Note that outside psychology, the least-squares model with component scores and loadings is often wrongly named factor model.

9 Settings with three components and with the PS equal to 0% are available as Online Resource 1.

10 The data can be accessed from the NCBI GEO database ( Nishimura et al. 2007), using accession number GSE7329. After personally contacting the corresponding author, we were informed that the data for the individuals GSM176586 (autism with FMR1FM, AU046707), GSM176589 (autism with FMR1FM, AU046708), and GSM176615 (control, AU1165305) were not correctly stored in the database. Therefore, the data for these individuals were not used in our analyses.
