# Supplementary material for: A Guide for Sparse PCA: Model Comparison and Applications
Source: Psychometrika. 2021 Jun 29;86(4):893–919. doi: 10.1007/s11336-021-09773-2 (PMC8636462; doi:10.1007/s11336-021-09773-2)
Supplement: Supplementary file 1 — Supplementary material 1 (pdf 439 KB) [file 11336_2021_9773_MOESM1_ESM.pdf]

A guide for sparse PCA: model comparison and applications

Abstract

PCA is a popular tool for exploring and summarizing multivariate data, especially those consisting of many variables. PCA, however, is often not simple to interpret, as the components are a linear combination of the variables. To address this issue, numerous methods have been proposed to sparsify the non-zero coefficients in the components, including rotation-thresholding methods and, more recently, PCA methods subject to sparsity inducing penalties or constraints. Here, we offer guidelines on how to choose among the different sparse PCA methods. Current literature misses clear guidance on the properties and performance of the different sparse PCA methods, often relying on the misconception that the equivalence of the formulations for ordinary PCA also holds for sparse PCA. To guide potential users of sparse PCA methods, we first discuss several popular sparse PCA methods in terms of where the sparseness is imposed on the loadings or on the weights, assumed model, and optimization criterion used to impose sparseness. Second, using an extensive simulation study, we assess each of these methods by means of performance measures such as Squared Relative Error, Misidentification Rate, and Percentage of Explained Variance for several data generating models and conditions for the population model. Finally, two examples using empirical data are considered.

Key words: Dimension reduction; Exploratory data analysis; High-dimension-low-sample-size; Regularization; Sparse principal components analysis.

1 Simulation Results Using 3 Components

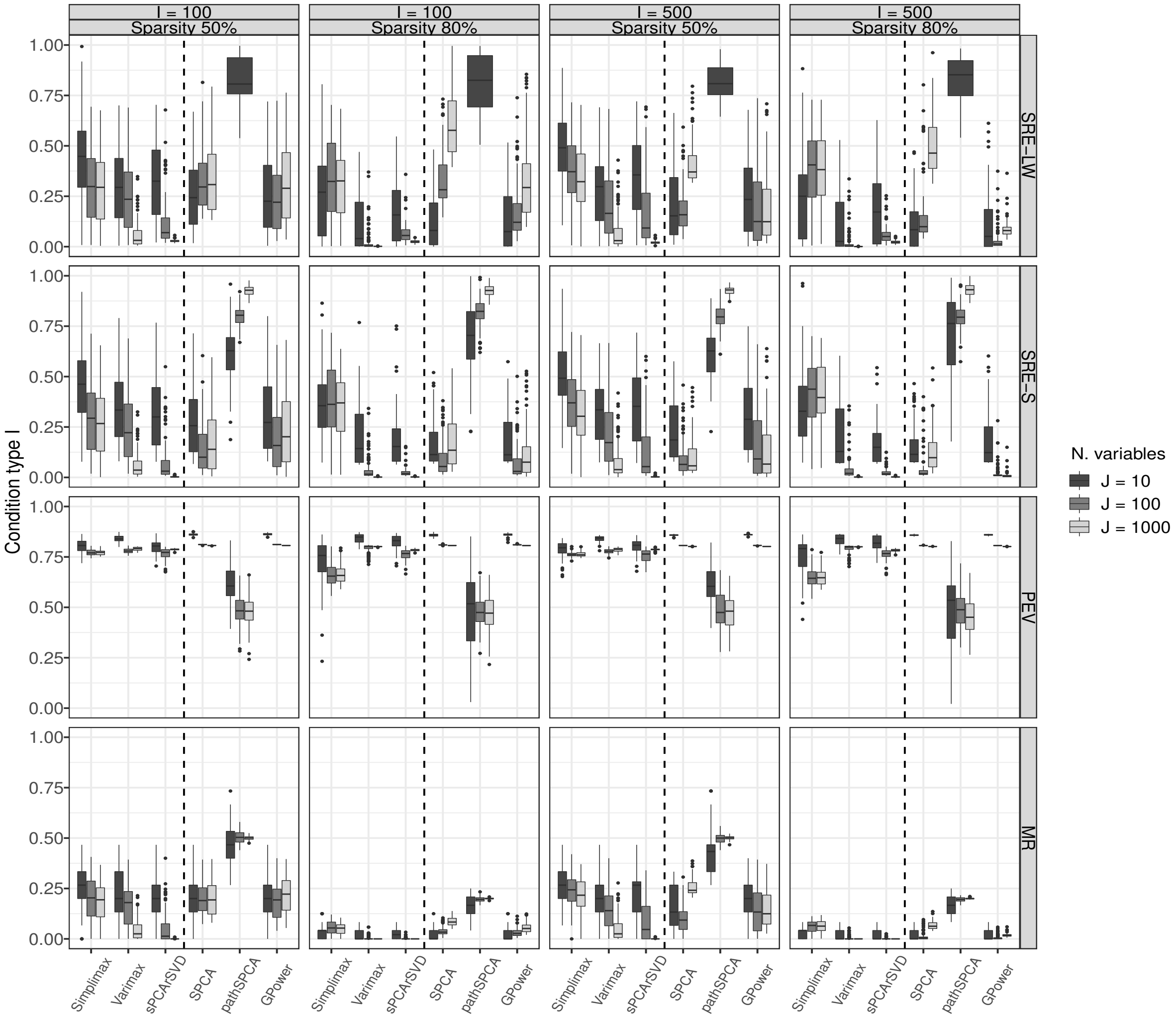

Figure 1.1: Matching sparsity: Boxplots of the performance measures in conditions with 80% of variance accounted by the model in the data and three components. Within each panel, a dashed line divides the boxplots for sparse loadings methods (at the left side of the dashed line) from those for sparse weights methods. The top row summarizes the squared relative error (SRE-LW) for the loadings (at the left of the dashed line) and weights (at the right of the dashed line), the second row the SRE-S for the component scores, the third row (PEV) the proportion of variance in the data explained by the estimated model, and, the bottom row, the misidentification rate (MR).

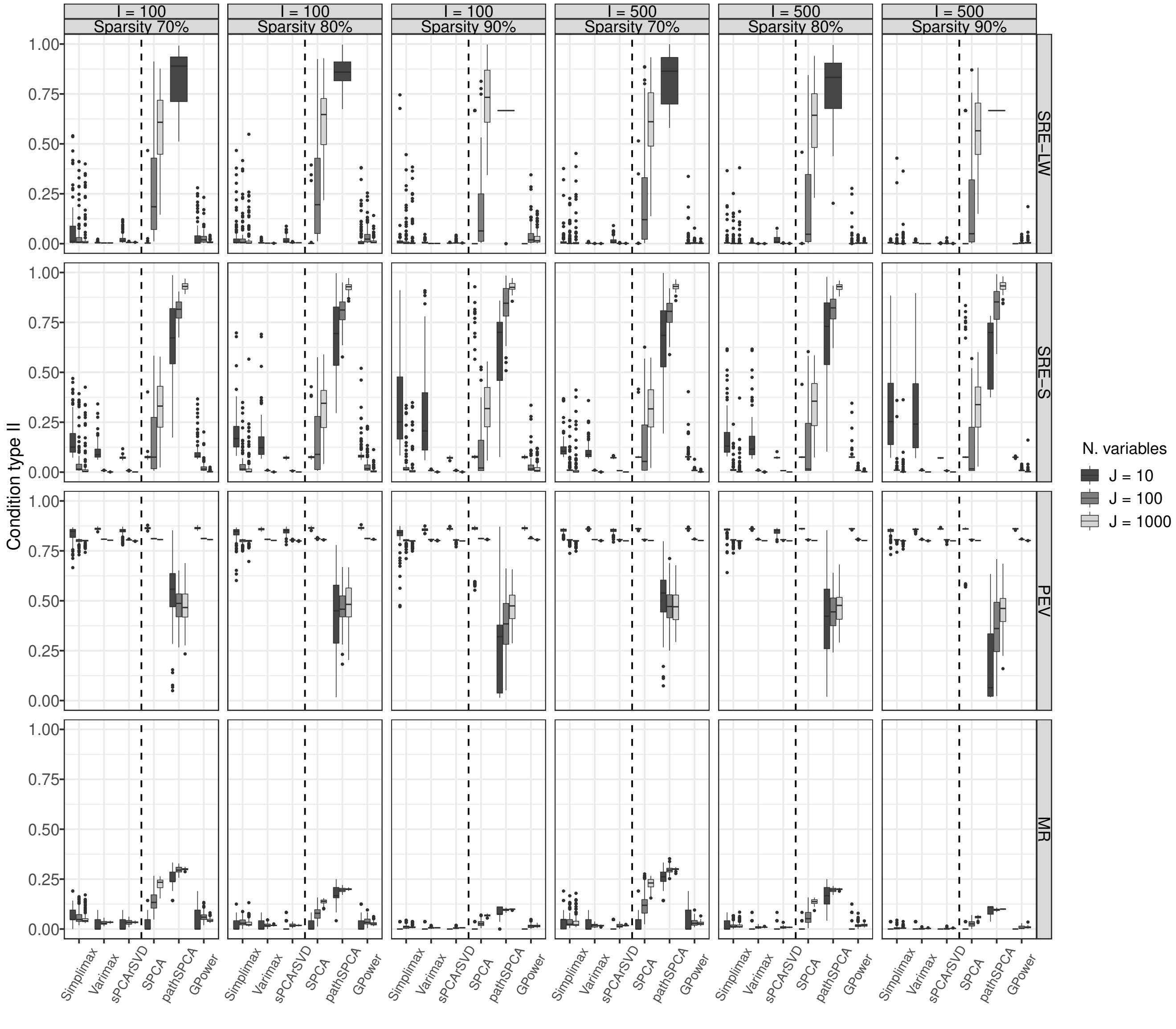

Figure 1.2: Double sparsity: Boxplots of the performance measures in conditions with 80% of variance accounted by the model in the data and three components. Within each panel, a dashed line divides the boxplots for sparse loadings methods (at the left side of the dashed line) from those for sparse weights methods. The top row summarizes the squared relative error (SRE-LW) for the loadings (at the left of the dashed line) and weights (at the right of the dashed line), the second row the SRE for the component scores, the third row (PEV) the proportion of variance in the data explained by the estimated model, and, the bottom row, the misidentification rate (MR).

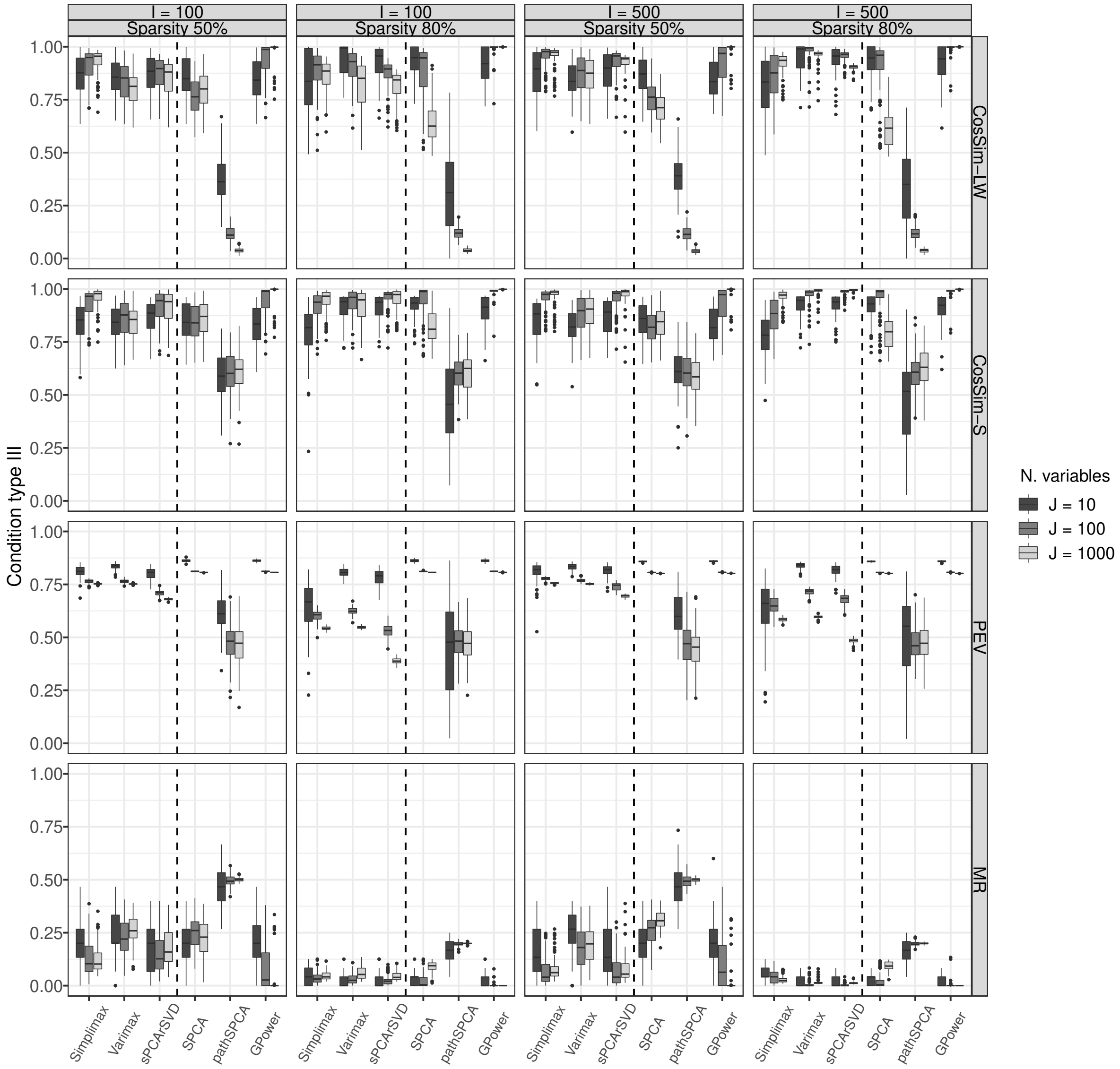

Figure 1.3: Miss-matched sparsity: Boxplots of the performance measures in conditions with 80% of variance accounted by the model in the data and three components. Within each panel, a dashed line divides the boxplots for sparse loadings methods (at the left side of the dashed line) from those for sparse weights methods. The top row summarizes the squared relative error (SRE-LW) for the loadings (at the left of the dashed line) and weights (at the right of the dashed line), the second row the SRE-S for the component scores, the third row (PEV) the proportion of variance in the data explained by the estimated model, and, the bottom row, the misidentification rate (MR).

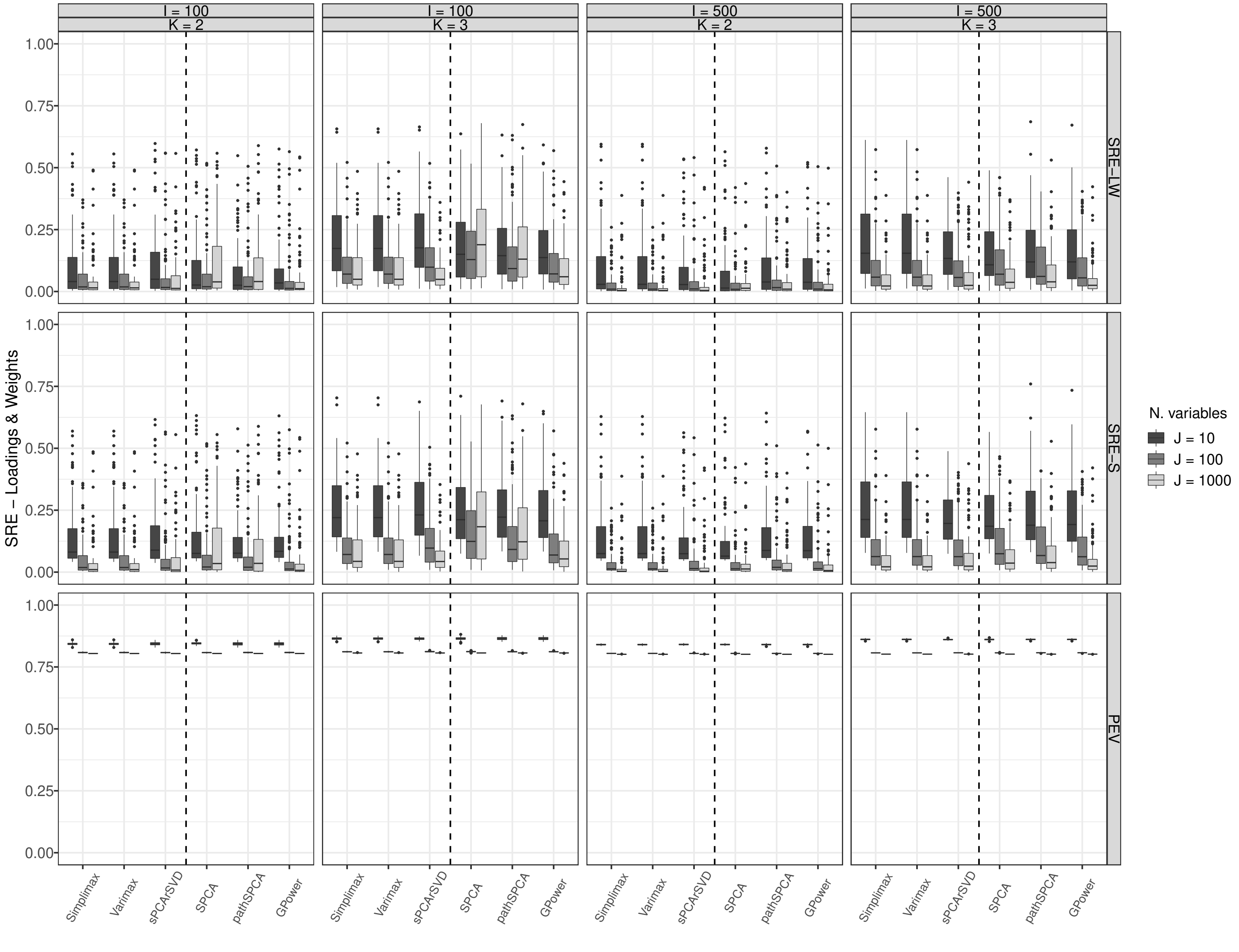

Figure 1.4: Ordinary PCA: Boxplots of the performance measures in conditions with 80% of variance accounted by the model in the data and 0% of sparsity. Within each panel, a dashed line divides the boxplots for sparse loadings methods (at the left side of the dashed line) from those for sparse weights methods. The top row summarizes the squared relative error (SRE-LW) for the loadings (at the left of the dashed line) and weights (at the right of the dashed line), the second row the SRE-S for the component scores, the third row (PEV) the proportion of variance in the data explained by the estimated model.
